# Inelastic Neutron Scattering Study of the Optically Excited State of MAPbBr_3_

**DOI:** 10.1021/acsomega.6c01172

**Published:** 2026-05-29

**Authors:** Kanming Shi, Hamish Cavaye, Rasmus Lavén, Maths Karlsson

**Affiliations:** † Department of Chemistry and Chemical Engineering, 11248 Chalmers University of Technology, Göteborg SE-412 96, Sweden; ‡ ISIS Pulsed Neutron and Muon Source, 120797 STFC Rutherford Appleton Laboratory, Chilton OX11 0QX, U.K.

## Abstract

Halide perovskites (HPs) have emerged as technologically appealing materials for a vast array of optoelectronic applications. However, fundamental questions surrounding the local structure and vibrational dynamics remain to be elucidated for these materials, especially regarding how they respond to the formation of photoexcited polarons. Here, in an inelastic neutron scattering (INS) study of the prototypical HP MAPbBr_3_ (MA = methylammonium, ${\mathrm{CH}}_{3}{\mathrm{NH}}_{3}^{+}$ ), we show that the formation of photoexcited polarons upon LED (light-emitting diode) illumination at 540 nm leads to changes in the INS spectrum. A comparison to INS spectra measured at different temperatures shows that the spectral change upon LED illumination originates from a change in the local coordination of the MA cations, rather than being only thermal in nature. This is primarily manifested as a change in the shape and intensity of the three strongest vibrational bands, located at ∼93, 110, and 295 cm^–1^, which are related to different MA vibrations coupled to vibrational modes of the PbBr_6_ octahedra. This new insight, together with the unique sensitivity of INS to hydrogen and its absence of vibrational selection rules, motivates further work utilizing *in situ* light illumination techniques in INS experiments as a route to develop a better understanding of the local structure and dynamics in HPs under operationally relevant conditions.

## Graphical abstract

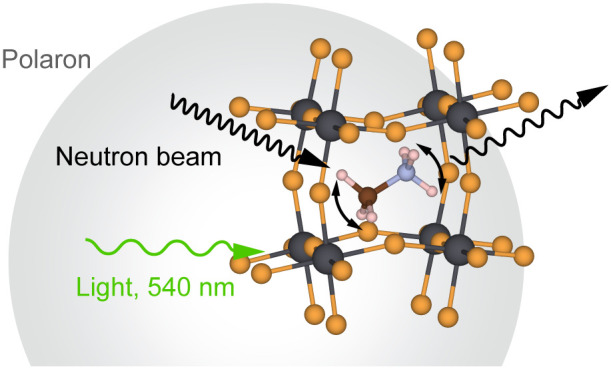

## 1 Introduction

HPs, of the form ABX_3_, where A is an organic cation, B is a metal cation, and X is a halide ion, are a unique class of photosensitive materials that have gained attention in recent years for their use in optoelectronic applications, such as solar panels and lighting.^1^ Upon photoexcitation, the electron–hole pairs typically form excitons, which may spontaneously separate into free electrons and holes (charge carriers) that may be collected to produce electricity, as in a solar cell, or which may recombine radiatively to emit light (photoluminescence), as in a LED.^1^ Because of the generally soft nature of the lattices of HPs,^2,3^ the photogenerated charge carriers or excitons can couple to phonons and molecular vibrational modes. This can give rise to local structural distortions^4,5^ and to polarons or self-trapped excitons, respectively, which subsequently lead to lower charge carrier mobility and influence the probability of charge recombination. However, the exact mechanisms of photoexcitation and deexcitation or other relaxation remain unclear.^6–8^

In ABX_3_ type HPs, the mechanism following photoexcitation is typically discussed in terms of the formation of a large polaron, which can protect the charge carrier from scattering and trapping, resulting in long charge carrier lifetimes and diffusion lengths.^9–11^ However, in addition to phonons, the reorientational dynamics of the organic cations are believed to play a role in this mechanism.^4,7,8^ In this regard, recent results from mid-infrared (IR) spectroscopy on the prototypical HP MAPbBr_3_ $\left(\mathrm{MA}={CH}_{3}{\mathrm{NH}}_{3}^{+}\right)$ provided strong support for polaron formation by observing the appearance of new vibrational modes, especially N–H stretching bands at around 2900 cm^–1^, upon photoexcitation.^12^ The appearance of new N–H stretching bands was hypothesized to result from a change in the strength of hydrogen-bonding interactions between MA and Br^–^ ions of the surrounding PbBr_6_ octahedra.^12^ Specifically, the change in hydrogen bonding resulted from local lattice distortion, related to the tilting of the PbBr_6_ octahedra, caused by the formation of photoexcited polarons.^12^ However, as the study was limited to the mid-IR region (≈800–4500 cm^–1^), any effects on the lower-frequency phonon modes or on the librational motions of the MA cations, which are sensitive to the degree of hydrogen-bonding interactions^13^ and are expected to show a strong effect upon polaron formation,^12,14^ were not studied.

In this work, we investigate the effect of light illumination at 540 nm, and thereby the formation of photoexcited polarons, on the phonon modes and MA librational modes in MAPbBr_3_. Differently from the mid-IR spectroscopy study reported in ref. 12, we use the INS technique, which is particularly suited to study low-energy motions and is sensitive to vibrational modes involving hydrogen. Previous INS studies show that MAPbBr_3_ exhibits several distinct bands, related to phonon modes and librational modes of the MA cation, well below 500 cm^–1^.^15,16^ However, it remains to be investigated how these modes, and hence the local structural properties of the material, are affected by light illumination. Such information is key to developing a fundamental knowledge of the mechanism of polaron formation in this and other HP materials. Furthermore, we discuss the perspectives for future research in this field.

## 2 Experimental Details

The INS experiment was performed on the indirect geometry spectrometer TOSCA at the ISIS Pulsed Neutron and Muon Source.^17^ The instrument provides an energy resolution of approximately 1% of the energy transfer. The sample, MAPbBr_3_ powder, was purchased from Xi’an Polymer Light Technology Corporation and was used as received. MAPbBr_3_ exhibits a cubic phase (space group *Pm*3̅*m*) for *T* > 220 K, a tetragonal phase (space group *I*4/*mcm*) for 145 ≤ *T* ≤ 220 K, and an orthorhombic phase (space group *Pnma*) for *T*  < 145 K.^18^ Room-temperature powder X-ray diffraction (XRD) analysis confirmed the cubic structure with no observable impurities; see Figure S1 in the Supporting Information (SI).

Approximately 2.5 g of MAPbBr_3_ powder was used for the experiment. Sample environment hardware for *in situ* light illumination has been developed for TOSCA.^19^ We used a new sample cell, specifically designed for the study of powder samples (Figure 1). The sample, a 0.5 mm thick layer of MAPbBr_3_ powder, is held between two quartz windows (5 cm in diameter) secured by an aluminum frame and sealed with indium wire. Two LEDs, each characterized by an emission wavelength of λ = 540 nm and a power of *P* = 0.94 W, are positioned on a metal bracket, mounted obliquely above the sample cell on opposite sides to illuminate the sample. The LEDs are located 40 mm from the cell center, resulting in an illumination distance range of approximately 30 to 60 mm across the sample surface. The LED photon energy is 2.296 eV and is chosen to be close to, but above, the bandgap of MAPbBr_3_ which is 2.28 eV at 50 K.^20^ All handling of the sample and the loading of the sample cell were performed in an Ar glovebox.

**Figure 1:**
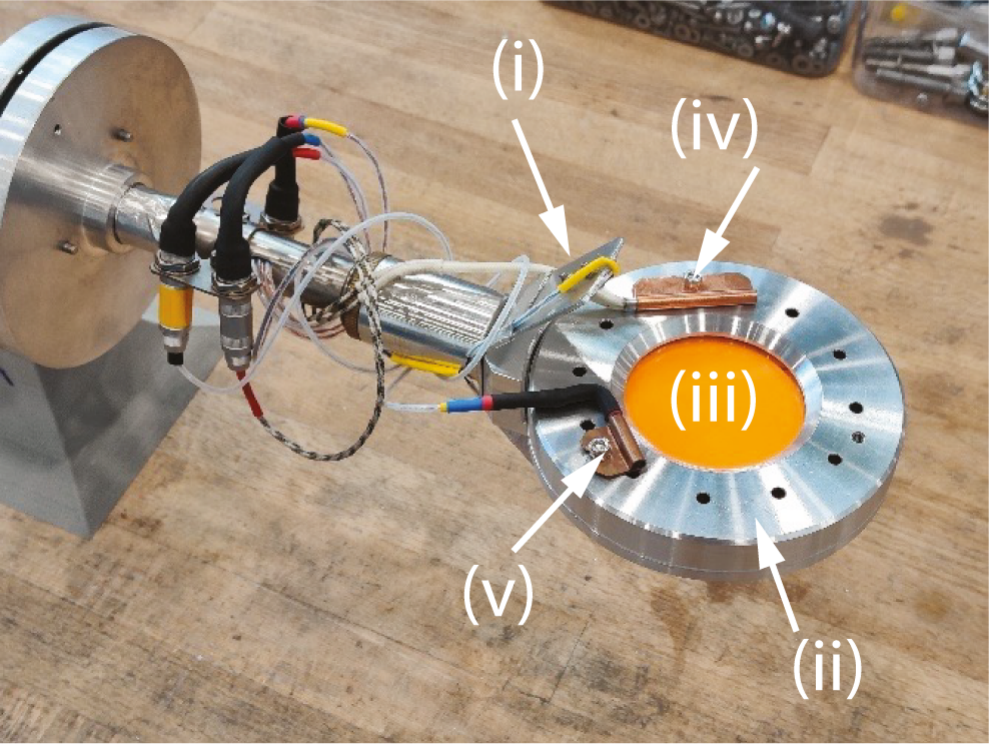
A photograph of the newly developed metal LED bracket (i) and illumination cell (ii) containing the MAPbBr_3_ sample (iii), for use on TOSCA. Also visible are the cartridge heater (iv) and temperature sensor (v) for thermal control.

The experiment commenced in the dark mode (LED off) at 45.0 K. Upon switching on the LEDs, heating by the LEDs caused the sample temperature to increase to 50.7 K. To differentiate optical effects from thermal effects, the temperature was subsequently maintained at 50.7 K for all further measurements. One LED on/LED off measuring cycle was performed under this isothermal condition. Consequently, all data presented in this work are from measurements in the orthorhombic phase of MAPbBr_3_. For a detailed experimental protocol, see Figure S2. The measured raw data were reduced in the Mantid Workbench^21^ and exported in ASCII format.

## 3 Results

### 3.1 INS Spectra Off and On Light Illumination

Figure 2 shows the INS spectra of MAPbBr_3_, as measured at 50.7 K, with the LEDs both off and on. As can be seen, the spectra are overall very similar to each other. The spectra are in excellent agreement with previous INS reports on the orthorhombic phase of MAPbBr_3_.^15,16^ Bands in the range of 10–65 cm^–1^ are related to octahedral twist motions, bands in the range of 65–120 cm^–1^ are related to nodding motions of the MA cation, bands in the range of 120–210 cm^–1^ are related to lurching motions of the MA cation, and the band at approximately 295 cm^–1^ relates to a C–N torsion motion of the MA cation.^15,16^ The three strongest bands, at around 93, 110, and 295 cm^–1^ are the ones showing the strongest effect upon photoexcitation by light illumination.

**Figure 2:**
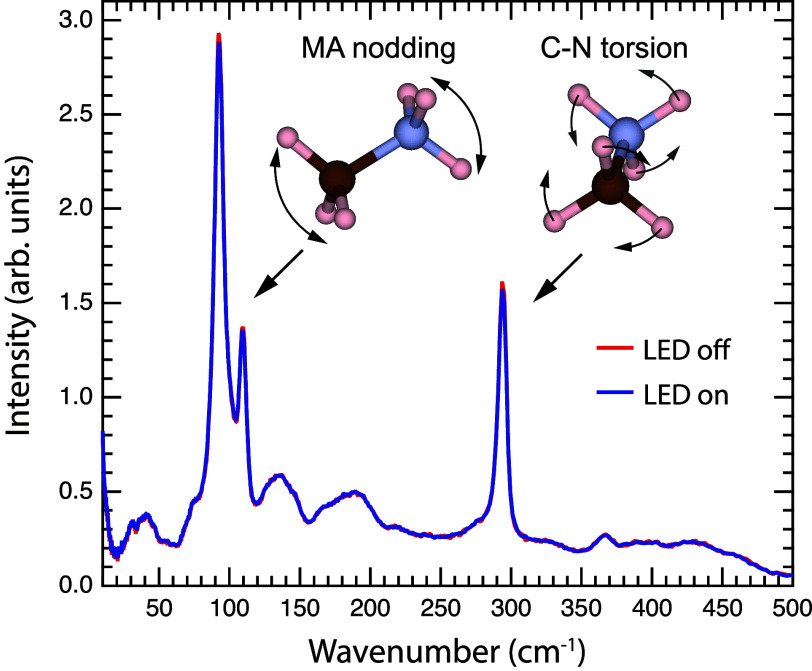
The INS spectrum of MAPbBr_3_, as measured at 50.7 K, is shown for LED off (red color) and LED on (blue color). Included in the graph are schematic illustrations of the MA nodding (65–120 cm^–1^) and C–N torsion (295 cm^–1^) motions. Light blue spheres represent nitrogen, brown spheres represent carbon, and light pink spheres represent hydrogen.

The spectral changes upon light illumination are further reflected in Figure 3, which shows the INS difference spectrum, obtained by subtracting the LED-on spectrum from the LED-off spectrum, both measured at *T* = 50.7 K. Although one can see that the spectral changes upon light illumination are undoubtedly small, a comparison of the INS spectra with the LED on and measured at different times (Figure S3) shows there is no sample degradation occurring due to light illumination, and the sample is robust. In addition to the changes in the INS spectrum upon light illumination, we observe insignificant changes in the elastic intensity (Figure 3a).

**Figure 3:**
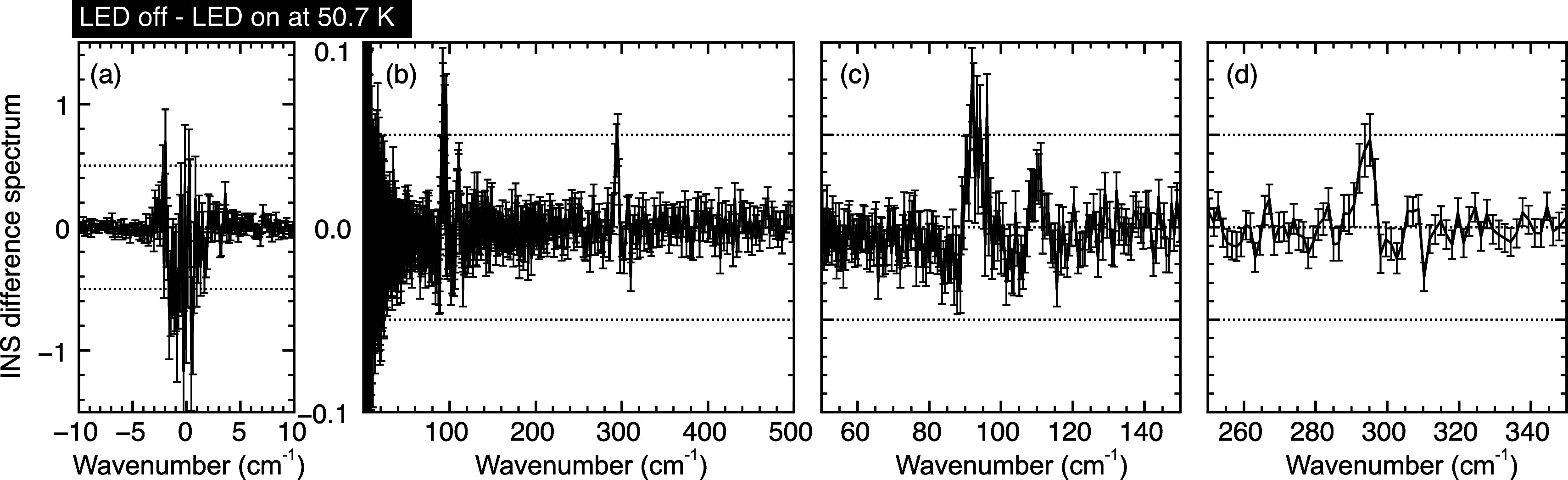
INS difference spectrum (LED off–LED on) of MAPbBr_3_, as measured at 50.7 K, is plotted over different energy regions: (a) −10 to 10 cm^–1^, (b) 10 to 500 cm^–1^, (c) 50 to 150 cm^–1^, and (d) 250 to 350 cm^–1^.

### 3.2 Variable Temperature INS Spectra

Figure 4 shows the INS difference spectra of MAPbBr_3_, as obtained by subtracting the LED-off spectrum measured at 50.7 K from that at 45.0 K. Qualitatively, this INS difference spectrum is very similar to the one shown in Figure 3 and, hence, exhibits similar changes in the three bands at around 93, 110, and 295 cm^–1^. This indicates that spectral changes due to light illumination are virtually the same, or at least similar, to those observed upon heating the sample from 45.0 to 50.7 K. However, in contrast to the effect of light illumination (Figure 3), heating has a stronger effect on the INS peaks and, additionally, the elastic peak intensity drops considerably. We speculate that the generally stronger features in Figure 4 as compared to Figure 3 may be a consequence of the fact that heating will have an effect on the entire sample, unlike the limited penetration depth of the light, whereas the reduction in elastic intensity reflects an increased population of vibrational modes.

**Figure 4:**
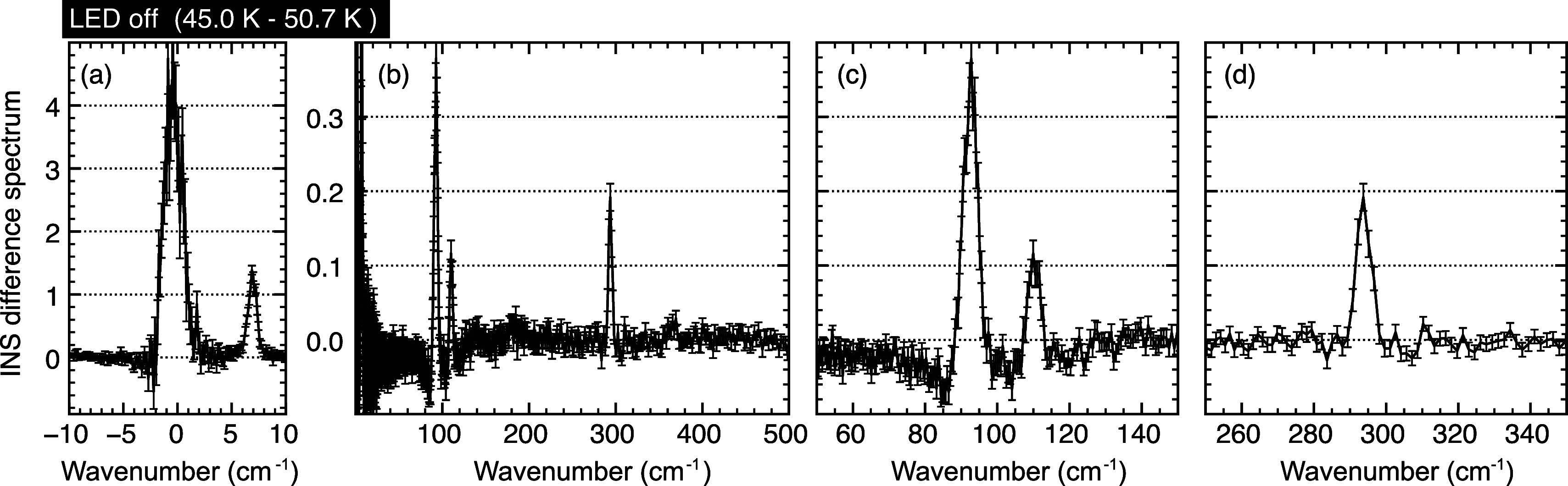
The INS difference spectrum (45.0–50.7 K) of MAPbBr_3_, as measured with the LED off, is plotted over different energy regions: (a) −10 to 10 cm^–1^, (b) 10 to 500 cm^–1^, (c) 50 to 150 cm^–1^, and (d) 250 to 350 cm^–1^. The peak at around 6.5 cm^–1^ is an instrumental glitch.

The large change in elastic intensity upon heating, together with no change in elastic intensity upon light illumination, is an important observation that suggests the formation of photoexcited polarons affects the local coordination of MA cations rather than simply giving rise to surface and near-surface heating. One should note, however, that the relative change in the elastic peak intensity is smaller than the relative change in the inelastic peak intensity. For the difference spectrum between the LED off at 45.0 and 50.7 K, the percentage change is about one-fifth of the inelastic peak change for the elastic peak. If we assume that the same ratio of change would also occur for the LED on run, it would mean a decrease in elastic intensity of about 0.4% for the LED on measurements at 50.7 K compared to the LED off at the same temperature. While relatively close, this is still above the statistical error of the measurements, again indicating that the LED on has another effect other than just local heating.

### 3.3 Peak-Fitting Analysis

For a detailed analysis of these bands, we performed a peak-fitting analysis over the range of 70–130 cm^–1^ and 260–330 cm^–1^, respectively. Figure 5 shows the results of the peak-fitting analysis to the INS spectra for (a, b) LED on at 50.7 K, (c, d) LED off at 50.7 K, (e, f) LED off at 50.0 K, and (g, h) LED off at 45.0 K. The spectra were fitted to a sum of four pseudo-Voigt functions (A, B, C, and D) to reflect the convolution of the vibrational peaks (Lorentzian line shape) with the instrumental resolution function (Gaussian line shape), and a background. The background—the same for all four spectra—consists of a linear line between 60 and 156 cm^–1^ and between 250 and 350 cm^–1^ as well as four Gaussian functions that approximate the INS intensity at the sides of the main peaks of interest. These background parameters, as well as the fraction between the Lorentzian and Gaussian components (*f*), were first determined by fitting the INS spectrum for LED off at 50.7 K. They were subsequently fixed during the fitting of the three other INS spectra. This allows for a comparison of the peak position, peak height, peak area, and fwhm of the four pseudo-Voigt functions between the four different INS spectra. The fitting parameters (Table S1) show no significant differences in peak position for any of the four peak-fitted components A, B, C, or D. However, a statistically significant change in peak height and fwhm can be seen for components A and C when comparing the LED on and LED off spectra at 50.7 K. For peak D, the peak height and fwhm also change in a similar manner; however, the change in fwhm is just within the error bar. It can be inferred, therefore, that while the spectral differences induced by the light illumination are small, they are statistically significant and larger than the uncertainty of the measurement. The components A, C, and D appear to broaden (lower peak height and wider fwhm with the same peak area) under illumination.

**Figure 5:**
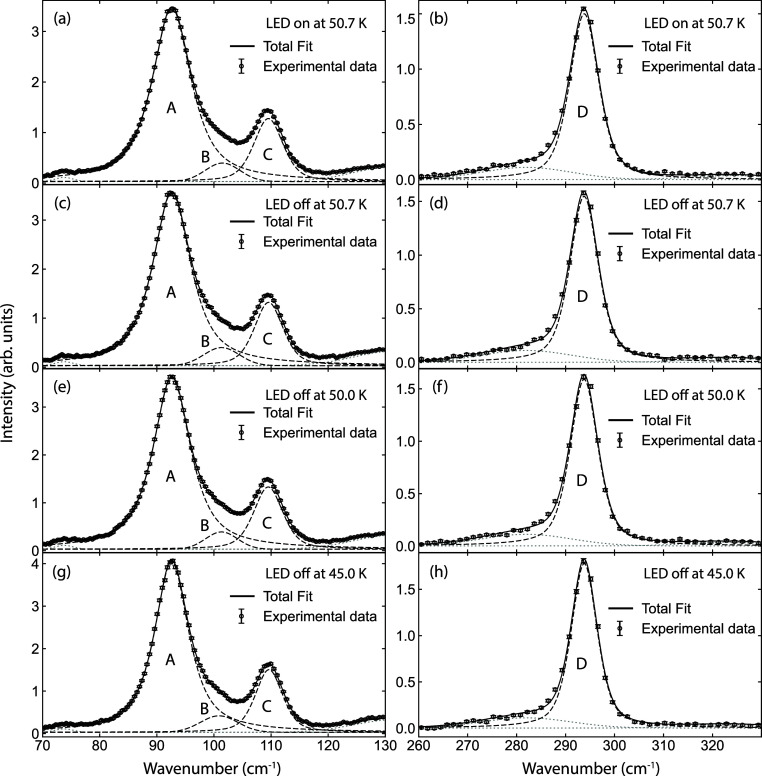
Peak fit to the INS spectra of MAPbBr_3_, as measured for (a, b) LED on at 50.7 K, (c, d) LED off at 50.7 K, (e, f) LED off at 50.0 K, and (g, h) LED off at 45.0 K.

## 4 Discussion

A key result is the clear difference between the LED-off and LED-on states on the INS spectrum, showing that the librational modes of the MA cation (ca. 93, 110, and 295 cm^–1^) are affected by photoexcitation and polaron formation. Since these librational modes are affected by the hydrogen-bonding interactions between MA and the Br^–^ ions of the surrounding PbBr_6_ octahedra,^13^ the change in these modes effectively originates from a change in the inorganic perovskite lattice and/or from a changing orientation of the MA molecule upon the formation of photoexcited polarons. This result is therefore in full agreement with the study reported in ref 12 that showed that the photoexcitation in MAPbBr_3_ leads to local lattice distortion mediated by the reorientation or “freezing” of the MA cations into other positions.

Nevertheless, the changes in the vibrational features upon the formation of photoexcited polarons are very small. This is primarily because the penetration depth of the light is very low, and so only a very small percentage of the sample is actually being illuminated. A single crystal of MAPbBr_3_ exhibits an absorption coefficient of around 1–5 × 10^4^ cm^–1^ at 540 nm and 300 K.^22,23^ This means that the penetration depth (here calculated as the depth at which the light intensity has decreased to 1%) is only about 1 μm. By taking into account that both sides of the sample are illuminated, this implies that only about 0.4% of the sample is being illuminated. Considering the larger effective surface area and the multiple light scattering effect of the powder sample (from its morphological roughness), the reabsorption effect,^24^ and the micrometer-scale exciton diffusion length in MAPbBr_3_,^25^ the proportion of the sample affected by the light-induced exciton–phonon coupling may extend beyond the optical penetration depth, and so this value of 0.4% can be considered a minimum value. Note that simply increasing the power of the light source to enhance the proportion of the illuminated sample is not ideal. As light absorption is exponential with penetration depth, a huge increase in power is required to illuminate more of the sample, which is not only inefficient but can also lead to damage of the sample. Instead, increasing the proportion of the sample surface that is illuminated, such as by the study of thin films, would greatly increase the percentage of the sample that is illuminated by light. However, this would require more sensitive neutron instrumentation.

The mismatch between the large sample volume required for INS and the low penetration depth of light represents a major challenge, not limited to MAPbBr_3_ but, in principle, to all materials. Future studies in this field may therefore involve the design of novel sample cells so that the proportion of the illuminated sample is enhanced. They may also take advantage of next-generation INS instruments and neutron sources, which will deliver a higher neutron flux at the sample position, enabling the study of smaller samples.

## 5 Conclusions

To conclude, we investigated the effect of the formation of photoexcited polarons on the INS spectrum and local structural properties of the orthorhombic phase of MAPbBr_3_. The INS measurements revealed differences, especially related to the librational modes of the MA cation (ca. 93, 110, and 295 cm^–1^), between the spectra measured with and without light illumination at 540 nm. The spectroscopic results support previous findings by mid-IR spectroscopy experiments, which suggest that light illumination leads to a local lattice distortion affecting the position and geometry of the MA cations within the perovskite lattice. However, the observed effects upon light illumination are undoubtedly very small, because only about 0.4% of the sample is actually being illuminated. Strategies for improving the percentage of the sample being illuminated by light have been discussed.

## Supplementary Material



## Notes

XRD Data and Analysis, INS Measuring Protocol, Additional INS Data (PDF)
