# Supplementary material for: Inelastic Neutron Scattering Study of the Optically Excited State of MAPbBr3
Source: ACS Omega. 2026 May 29;11(22):32542–7. doi: 10.1021/acsomega.6c01172 (PMC13261460; doi:10.1021/acsomega.6c01172)
Supplement: Supplementary file 1 [file ao6c01172_si_001.pdf]

Supporting Information:

Inelastic Neutron Scattering Study of the Optically Excited State of $MAPbBr_3$

Kanming Shi,[a] Hamish Cavaye,[b] Rasmus Lavén,[a] and Maths Karlsson*[a]

[a] *Department of Chemistry and Chemical Engineering, Chalmers University of Technology, SE-412 96 Göteborg, Sweden*
[b] *ISIS Pulsed Neutron and Muon Source, STFC Rutherford Appleton Laboratory, Chilton, OX11 0QX, UK*
*Email: maths.karlsson@chalmers.se

Contents

S1 XRD Data and Analysis **S2**

S2 INS Measuring Protocol **S2**

S3 Additional INS Data **S2**
S3.1 Comparison of INS Spectra Measured at Different Times S2
S3.2 Peak-Fitting Parameters S3

S1 XRD Data and Analysis

The XRD measurements were performed using a Bruker D8 Discover with a Cu target (K_α = 1.5406 Å) at room temperature, at Chalmers University of Technology. Approximately 100 mg of finely ground $MAPbBr_3$ powder was used for the measurements and placed on a Si zero-background sample holder. The XRD data are shown in Fig. S1.

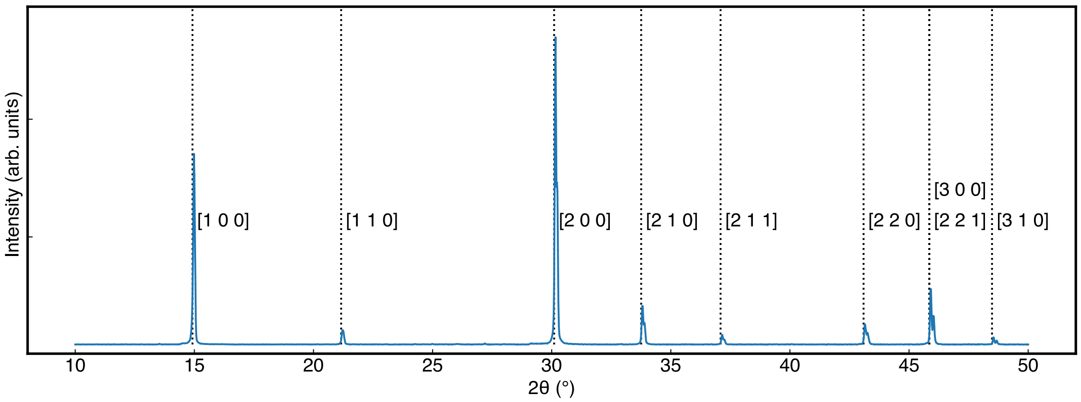

Fig. S1 XRD data for $MAPbBr_3$. The dashed lines represent the calculated peak positions labeled by Miller indices from ref. [S1].

S2 INS Measuring Protocol

Fig. S2 illustrates the experimental protocol. The LED on and LED off data reported in the main text were obtained by integrating signals over the green and gray shaded regions, respectively, during which the temperature was maintained at 50.7 K. For comparison, reference datasets were also collected under dark conditions (LED off) at 45.0 and 50.0 K.

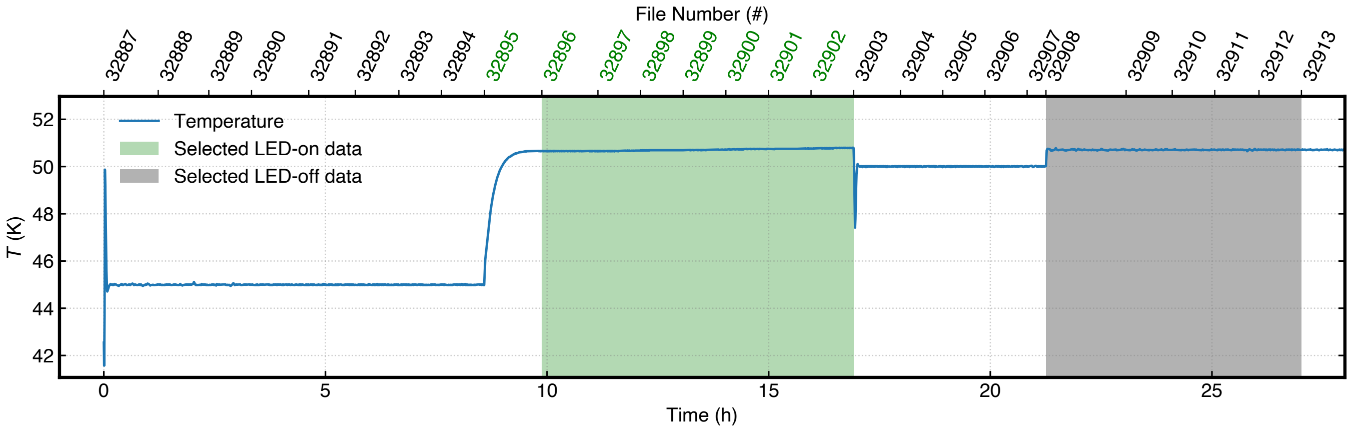

Fig. S2 Experimental protocol, showing the temperature of the $MAPbBr_3$ sample as a function of time from the start of the experiment (bottom) and measurement file number (top).

S3 Additional INS Data

S3.1 Comparison of INS Spectra Measured at Different Times

Fig. S3 compares the INS difference spectrum of $MAPbBr_3$, as obtained from the two measuring cycles. As can be seen, the INS difference spectrum is (within error) practically featureless, over the whole spectral range as investigated here. This suggests that

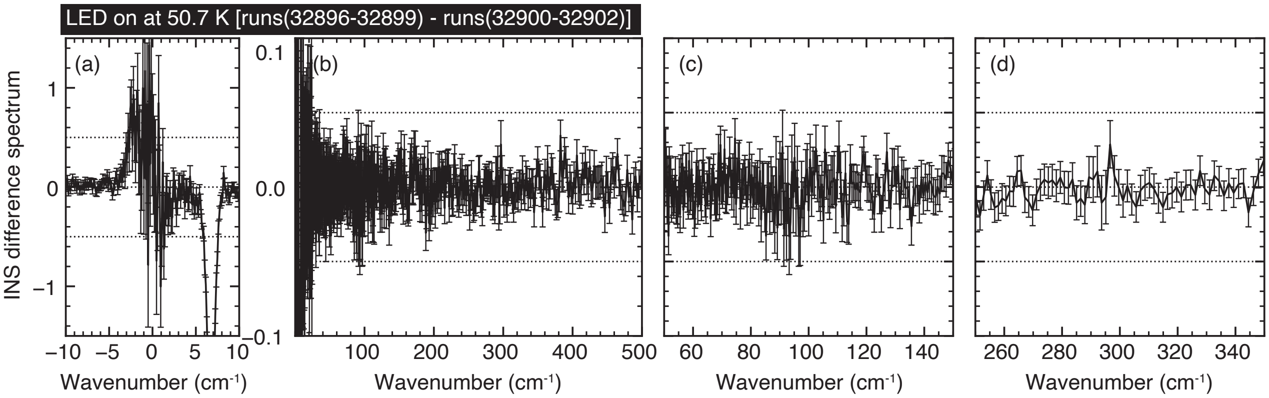

Fig. S3 INS difference spectrum, as derived from the differences between different runs with the LED on at 50.7 K, plotted over different energy regions: (a) -10 to 10 cm^{-1}, (b) 10 to 500 cm^{-1}, (c) 50 to 150 cm^{-1}, and (d) 250 to 350 cm^{-1}. The peak at around 6.5 cm^{-1} is an instrumental glitch.

the structure of the sample is preserved upon the light illumination, *i.e.* that the sample does not degrade with time under light exposure.

S3.2 Peak-Fitting Parameters

Table S1 Compilation of peak fitting parameters.

Function	Condition	Peak position (cm^{-1})	Peak height	Peak area	FWHM (cm^{-1})
Pseudo-Voigt	50.7 K LED on	92.62 ± 0.01	3.43 ± 0.01	41.07 ± 0.16	8.11 ± 0.04
$f = 0.86$	50.7 K LED off	92.62 ± 0.01	3.53 ± 0.01	41.15 ± 0.18	7.91 ± 0.04
(A)	50.0 K LED off	92.63 ± 0.01	3.59 ± 0.01	41.40 ± 0.16	7.82 ± 0.04
	45.0 K LED off	92.60 ± 0.02	4.03 ± 0.02	42.29 ± 0.25	7.12 ± 0.04
Pseudo-Voigt	50.7 K LED on	101.49 ± 0.10	0.37 ± 0.01	2.77 ± 0.15	7.12 ± 0.33
$f = 0.00$	50.7 K LED off	101.34 ± 0.11	0.37 ± 0.01	2.86 ± 0.16	7.29 ± 0.36
(B)	50.0 K LED off	101.32 ± 0.10	0.36 ± 0.01	2.54 ± 0.13	6.60 ± 0.31
	45.0 K LED off	100.90 ± 0.15	0.39 ± 0.01	3.16 ± 0.22	7.67 ± 0.48
Pseudo-Voigt	50.7 K LED on	109.53 ± 0.03	1.25 ± 0.01	9.74 ± 0.10	6.06 ± 0.06
$f = 0.54$	50.7 K LED off	109.57 ± 0.03	1.29 ± 0.01	9.86 ± 0.10	5.92 ± 0.06
(C)	50.0 K LED off	109.50 ± 0.03	1.30 ± 0.01	10.11 ± 0.09	6.02 ± 0.06
	45.0 K LED off	109.63 ± 0.03	1.47 ± 0.01	10.77 ± 0.12	5.66 ± 0.06
Pseudo-Voigt	50.7 K LED on	293.81 ± 0.04	1.50 ± 0.02	13.94 ± 0.13	7.01 ± 0.09
$f = 0.61$	50.7 K LED off	293.78 ± 0.04	1.55 ± 0.02	14.01 ± 0.14	6.84 ± 0.09
(D)	50.0 K LED off	293.78 ± 0.04	1.59 ± 0.02	14.20 ± 0.16	6.74 ± 0.10
	45.0 K LED off	293.80 ± 0.04	1.78 ± 0.02	14.86 ± 0.16	6.31 ± 0.09

References

[S1] A. Jaffe, Y. Lin, C. M. Beavers, J. Voss, W. L. Mao and H. I. Karunadasa, High-Pressure Single-Crystal Structures of 3D Lead-Halide Hybrid Perovskites and Pressure Effects on their Electronic and Optical Properties. *ACS Central Science*, 2016, **2**, 201–209.
